# A laboratory-based test procedure for the investigation of slaking-induced changes in geotechnical properties of tailing dam embankment materials

**DOI:** 10.1016/j.heliyon.2024.e26489

**Published:** 2024-02-19

**Authors:** Chaminda Gallage, Shiran Jayakody, Tharindu Abeykoon, Dareeju Biyanvilage, Jay Rajapakse

**Affiliations:** a School of Civil and Environmental Engineering, Queensland University of Technology, Australia; b Worley Limited, Australia

**Keywords:** Tailings storage facility, Rockfill materials, Slaking, Drying-wetting cycles, Shear strength, Permeability characteristics

## Abstract

Slaking is a process of material parameters alteration resulting from wetting-drying cycles, changes in overburden stress, and chemical interactions. Tailings Storage Facilities (TSF) constructed with materials prone to slaking may experience breaches, especially during the post-closure period, due to the deterioration of shear strength and permeability characteristics. Rockfill materials, particularly those containing clay components, can undergo various forms of crack formation, leading to disintegration as a result of wetting-drying cycles, stress increments, and intense compaction. However, there are currently limited methodologies available for replicating such material alterations on a laboratory scale. Therefore, a new large-scale laboratory testing approach has been designed to simulate variations in wetting-drying cycles, humidity, and overburden pressure, enabling the prediction of the slaking potential of TSF construction materials. This novel methodology replicates field drying-wetting cycles and variations in humidity and overburden stress in a controlled environment, allowing for the estimation of the deterioration of shear strength and permeability characteristics in rockfill materials.

## 1 Introduction

Tailings Storage Facilities (TSFs); used to manage the fine-grained wastes or tailings produced from extraction, beneficiation, or concentration processes, can be constructed using upstream, downstream, and centreline techniques depending on the types of waste produced from the mining operations [[1], [2], [3]]. Tailings, which exhibit chemical similarities to the parent materials but have undergone physical alterations during processes such as crushing, grinding, flotation, cyanidation, or acid leaching, are primarily water-based. As a result, they are typically deposited in Tailings Storage Facilities (TSFs) in the form of slurries, whether submerged (subaqueous) or in exposed (subaerial) conditions [4,5]. Even though the TSFs contained a significant amount of water storage, unlike water storage facilities (WSF), the selection of site and construction materials are constrained by the mining operations in addition to the common factors including topography, rainfall intensity, seismicity, and consequence category [[6], [7], [8]].

Earth and rock waste materials are being typically used to continuously construct the TSFs within the life of the mine to accommodate the increasing tailings volume [9]. However, unlike the WSFs, the best construction materials can't be accommodated to construct the TSF embankments due to the cost feasibility [10]. The earthfill and rockfill materials used to construct the TSF embankments are, therefore, prone to deteriorate by their original characteristics during operational and especially closure periods as a consequence of the variations of the wetting-drying cycles, humidity, and overburden pressure [[11], [12], [13]]. This deterioration process; known as the material slacking, can potentially alter the gradation and composition of the construction materials, which ultimately degrade the strength and permeability characteristics [14,15]. Deterioration of the permeability characteristics can potentially increase the saturation levels of the construction materials which can increase the probability of static liquefaction susceptibility and dam breach [16,17].

The stability of Tailings Storage Facility (TSF) embankments is contingent on the construction methods employed and the inherent characteristics of the tailing deposits. Failures in TSF embankments can be initiated by seepage-induced internal erosion, which manifests as excessive internal pore-water pressure or piping within the embankments [18]. Soft and saturated tailings pose a considerable hazard, especially when the material is saturated to the extent that it could lead to the development of a mud flood or mudflow in the event of a hypothetical dam breach [19]. The complexity of potential failure mechanisms in tailings dams often precludes their identification through simple investigative methods. A review of historical tailings dam failures has revealed two distinct mechanisms in which the strain softening behaviour of the material plays a pivotal role. The first mechanism pertains to the progressive development of failure in a weak soil layer within the dam foundation [20,21]. The second mechanism is associated with the static or dynamic liquefaction of loose tailings material at a critical state [22].

TSF embankment failures have been documented since 1915 [23] and out of 258 TSF embankment failures, approximately 22% are related to slope instability [24,25]. However, the attribution of slacking of the construction materials to these failures has not been examined. It is predicted that the aging TSF embankment could be the predominant hazard type for future dam breaches [26,27] and due to complexities associated with materials slacking, limited investigations have been conducted to date for evaluating the slacking potential of the construction materials. The currently established slaking tests, namely the jar slake test, slake index test, and slake durability test, have limitations in replicating the authentic triggering factors of slaking. These drawbacks become particularly apparent when attempting to analyse samples extracted from complex environments like Tailings Storage Facility (TSF) embankment materials, given the intricacies of the slaking process within such environments. Notable deficiencies associated with these conventional tests include [28,29]:

• Inadequate Sample Size Representation: The sample sizes required for each of these conventional tests are notably small, often comprising just a few grams of material. Consequently, these sample sizes may insufficiently reflect the complexity of in-situ materials. For instance, a minute piece of highly weathered material might be used to represent an entire section, which does not adequately encapsulate the true material diversity.
• Limited Testing Duration: Slaking is a time-dependent process that necessitates an extended duration to effectively initiate the slaking process. Conventional tests typically run for only a few hours, which may not suffice to induce the comprehensive slaking conditions required for robust analysis.
• Simulation of Real Slaking Conditions: Precise estimation of the slaking characteristics of materials demands that the testing samples be subjected to authentic slaking conditions. These conditions include stress increment, wetting and drying cycles, temperature variations, and more. Existing test methods often fall short in fully simulating these diverse slaking conditions.
• Influence of the Test Performer: The effectiveness and reliability of conventional test methods are further complicated by the significant influence of the test performer. Methodologies for these tests often rely on individual techniques, leaving room for variation. For instance, the assessment of sample category in the jar slake test or the washing methods applied to samples in the slake index tests lack standardized procedures and are contingent upon the preferences and practices of the test performer.

The recognized limitations of these conventional slaking tests underscore the need for the development of more comprehensive and standardized methodologies that align with the complex reality of slaking processes in diverse environments, such as TSF embankment materials. A novel laboratory-scaled testing approach has been, therefore, developed in this study to evaluate the slacking potential of the TSF construction materials. The experimental methodology was systematically devised to encompass all potential triggering factors contributing to the slaking process. Ample duration was allocated to ensure the accurate simulation of a variety of test conditions. The specimens underwent continuous exposure to an overburden pressure, analogous to the conditions present in a substantial stratum of the initial TSF embankment from which the materials were sourced. Following the overburden simulation, the specimens were meticulously saturated to accurately replicate the actual field water saturation process. In order to further replicate authentic environmental influences, the specimens were subjected to controlled weathering conditions within an environmental chamber. This environmental chamber was carefully regulated to maintain specific temperature and moisture parameters, thereby inducing wetting-drying cycles in accordance with the desired humidity thresholds. The field drying-wetting cycles and variations of the humidity and overburden stress can be replicated in this proposed testing approach which will, therefore, help TSF designers and mine operates to accurately predict the deterioration of the construction materials and its impacts on the stability of the TSF embankments. Further, the geotechnical properties of the slaked materials were investigated, and emphasised the significance of evaluating the changes in the properties to estimate the effect of slaking process.

## 2 Test material

Rockfill materials, which is predominantly sourced from siltstone, were obtained from a downstream constructed TSF embankment in Laos. The physical properties of the test materials were determined as per Australian standards and presented in Table 1.

**Table 1:** Physical properties of rockfill test material.

Parameter	Value
Grain size Distribution	% Finer than 4.75 mm > 18.69%
	USCS - Poorly graded gravel (GP)
Atterberg Limits	LL - 23.43 %
	PL - 12.17 %
Specific gravity	Passing the 2.36 mm sieve - 2.66
	Retained on 2.36 mm sieve - 2.67
Hydraulic conductivity	6.49E-05 m/s

Note: USCS - Unified Soil Classification System, LL - Liquid limit, PL – Plastic limit.

## 3 Sample preparation and testing program

**Fig. 1:**
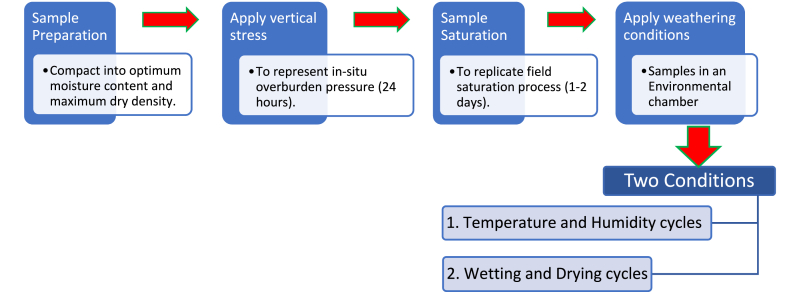
The process of simulating the triggering actions of slaking.

The slaking process disrupts the bonds and, breaks the specimens due to the overburden pressure, immersion, wetting and drying cycles. Therefore, a comprehensive experimental procedure was developed to replicate all potential slaking-inducing mechanisms within a controlled laboratory setting. The procedural stages are graphically depicted in Fig. 1. Subsequently, the testing methodology was expanded to determine the physical properties of slaked materials thereby, to reveal the slaking repercussions.

**Fig. 2:**
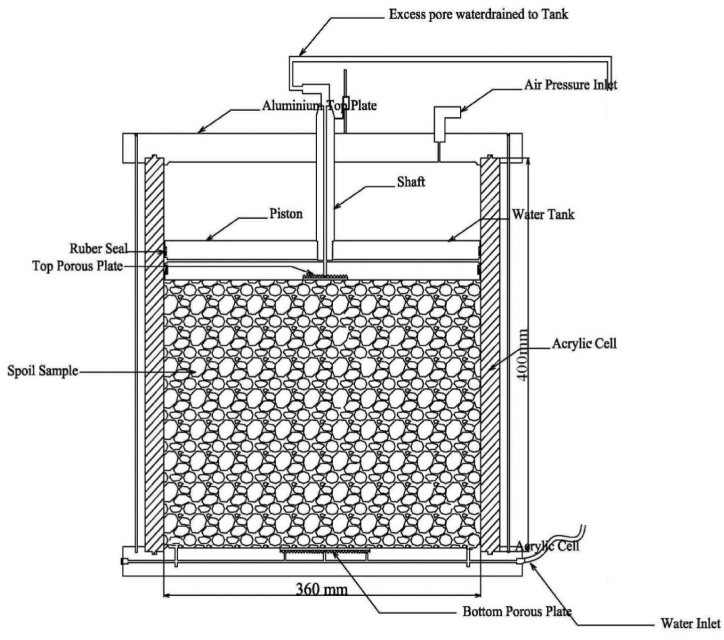
Schematic diagram of a slaking cell.

**Fig. 3:**
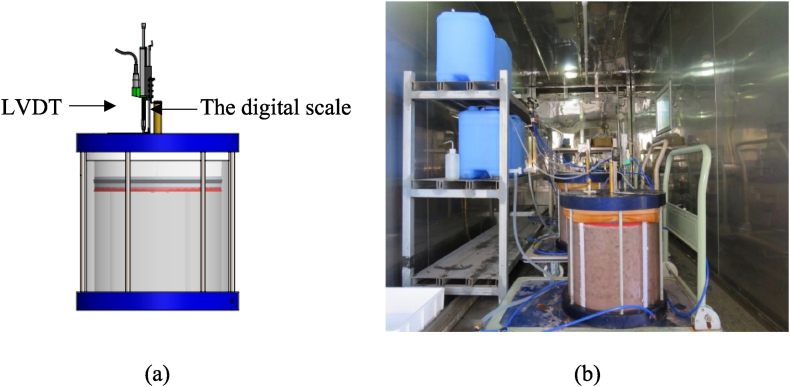
(a) LVDT and digital scale attachment on slaking cell (b) Setup inside the environmental chamber before initiation of slaking process.

Samples were prepared in large scale cylindrical cells with an internal diameter of 360 mm and materials were compacted up to 320 mm height as shown in Fig. 2. Large-scale samples effectively capture the intricate complexities of the slaking process, resulting in a more accurate representation of soil behaviour during simulated slaking. This approach enhances the comprehension of how materials react to the factors that initiate slaking. Four Samples were prepared in slaking cells to achieve 2.13 g/cm^3^ of maximum dry density and 7% of optimum water content. Each slaking cell was subjected to 925 kPa of effective overburden pressure, simulating effective overburden pressure of 900 kPa on the compacted materials and 25 kPa to overcome the friction between the piston and cell wall. The 900 kPa effective overburden pressure represents an approximately 40–45 m thick layer of the original TSF embankment where the materials were obtained. A Linear Variable Displacement Transducer (LVDT) and a digital scale were attached to the loading plate of each cell as shown in Fig. 3 (a) to measure volume change and any sudden collapse of the materials during the slaking process. The range and accuracy of the LVDT are 50 mm and ± 0.05% respectively. The slaking cells with the compacted materials were left over for 24 h with vertical stress to ensure uniform pressure distribution on the compacted samples. Samples were then saturated for 1–2 days to replicate the field water saturation process. Seepage poses a potential threat to the stability of Tailings Storage Facility (TSF) embankments, particularly in the context of soft and saturated materials that can trigger disintegration during the slaking process. Consequently, samples were meticulously saturated for a period of two days. Next, the four slaking cells were placed in an environmental chamber as shown in Fig. 3(b) to simulate environmental weathering conditions under two test conditions.

• Test condition 1 – two slaking cells were subjected to temperature and humidity cycles.
• Test condition 2 – two slaking cells were subjected to drying and wetting cycles.

### 3.1 Test condition 1

**Fig. 4:**
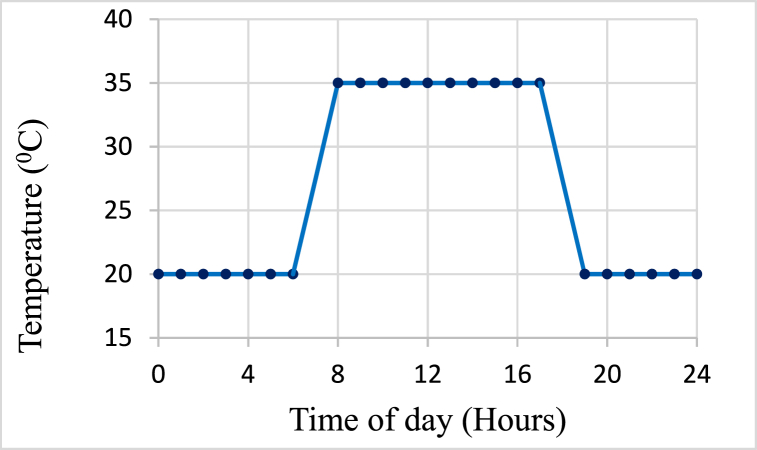
Daily temperature cycle in the environmental chamber during the slaking process.

Two slaking cells were drained out for 1–2 days to desaturate the samples, then, all drained valves were closed for six months. Slaking is a process that unfolds gradually over an extended period, influenced by factors such as weathering and seasonal variations. Consequently, an experimental setup was designed to conduct the test over a duration of six months. This extended timeframe aims to closely replicate natural conditions, ultimately facilitating a more precise simulation of in-situ behaviour. During the six months, these cells were subjected to daily temperature cycles (i.e., Slaking by temperature - Slaked by T). Fig. 4 shows the adopted daily temperature variations in the environmental chamber, replicating the in-situ conditions in TSF embankment in Laos where the samples were obtained. Furthermore, the humidity in the environmental chamber was maintained between 70 % and 90 % throughout the six months period. At the end of the six months, two cells were fully saturated before depressurizing to evaluate the post-collapse potential of slaked materials. The slaked materials of these two cells were then used to investigate the effect of slaking actions on basic classification, shear strength, and permeability properties.

### 3.2 Test condition 2

The remaining two cells were subjected to fortnightly drying and wetting cycles for six months with the overburden effective pressure of 925 kPa and daily temperature variations (i.e., Slaking by temperature and rainfall - Slaked by T & R). The compacted samples in the cells were fully saturated for the wetting cycle for a week and drained out on the 7th day to start the drying cycle. The conditions of the drying cycle were similar to the “Test condition 1” and were consistently maintained for a week, with humidity level maintained between 70 and 90 %. Following this one-week drying cycle, the wetting cycle commenced and was sustained for the next seven days by continuously introducing a controlled water flow through the sample under a constant head. Daily temperature variation was similar to the drying cycle during the wetting cycle. Fortnight wetting and drying cycles were consecutively applied for six months periods (13 wetting-drying cycles) subsequently, the effect of slaking on the geotechnical properties was determined as per the “test condition 1” at the end of the six months.

### 3.3 Physical properties-before and after slaking

The slaked materials in two test conditions were tested to determine basic classification, shear strength, and permeable properties to evaluate the impact of temperature, humidity, and rainfall variations on the particle degradation of rock-fill material. Fresh and, compacted materials (before slaking) were also subjected to the above tests to compare the effect of slaking process.

Physical properties were primarily determined to evaluate the effectiveness and influence of the slaking process on the test materials. PSD and Atterberg limit tests were conducted to determine the physical properties. Moreover, PSDs were conducted on sheared compacted specimens to assess the potential breakage of slaked materials when they were subjected to shearing.

Shear strength properties, are key indicators of soil stability and, slaking of materials can diminish these properties, making the soil structure more vulnerable to erosion, landslides, or structural instability. Therefore, direct shear test was conducted before and after the slaking process using a large direct shear apparatus (300 mm × 300 mm x 185 mm (height)), and samples were compacted into the shear box to achieve 2.13 g/cm^3^ dry density and 7 % gravimetric water content. Three identical samples were sheared in each test under constant vertical stresses of 300, 600, and 900 kPa to determine the shear strength parameter of slaked materials. Conducting three direct shear tests spanning a broad vertical stress range, from 300 to 900 kPa, enables the prediction of more precise failure envelopes based on the results of these tests. Slaking can alter the pore structure and fabric of soil, affecting its permeability characteristics. Variation in permeability can impact groundwater flow, drainage, and contaminant transport thus, it is vital for the effective management of water resources and the safeguarding of the environment in areas where materials susceptible to slaking are prevalent. A constant head permeability apparatus was used to determine the hydraulic conductivity of the fresh, compacted, and slaked materials. The permeability cell has an internal diameter of 150 mm, and materials was filled up to the height of 165 mm. The soil was compacted into the permeability cell to achieve 2.13 g/cm^3^ dry density and 7 % gravimetric water content. The test was conducted according to the AS 1289.6.7.1–2001.

## 4 Testing results

### 4.1 Mineralogy of materials

Samples from fresh, slaked by temperature and slaked by both temperature and rainfall were subjected to XRD analysis, and the corresponding results are presented in Table 2, Table 3. The powder XRD patterns show the presence of crystalline phases, and the results illustrate that there is no significant difference in compositions of the material and mineral compounds.

**Table 2:** Summary of phase abundances (nominal absolute weight percentage).

Phase	Fresh	Slaked by T	Slaked by T & R
Quartz	23.0	28.5	25.2
Hematite	3.6	3.2	3.1
Calcite	14.8	15.9	15.9
Dolomite	5.5	3.5	5.9
Plagioclase	23.5	25.9	27.2
K-Feldspar	1.4	0.5	0.9
Chlorite	3.2	4.0	3.8
Amorphous	3.0	4.1	4.5

**Table 3:** Summary of Fine fraction (clay phases) identifications (qualitative, nominal).

Phase	Fresh	Slaked by T	Slaked by T & R
Chlorite	Major	Major	Major
Smectite	Trace	Trace	Minor
Illite/mica	Abundant	Abundant	Abundant

It should be noted that during the sample analysis, the known addition of corundum facilitated to reporting the absolute phase abundances of the modelled phases. The sum of the absolute abundances was subtracted from 100% (weight percentage) to obtain the residuals (i.e. non-diffracting/unidentified, also known as “amorphous”). The residual represents the unexplained portion of the pattern: it may be non-diffracting content but could also contain unidentified phases and the error from poorly modelled phases. It was the least accurate measure as its error was the sum of the errors of the modelled phases.

Past research studies have revealed that rock materials containing Smectite, Montmorillonite and Pyrite minerals are extensively prone to disintegrate when they are exposed to triggering actions of slaking [15,30,31]. The mineralogy of the tested materials in this study was mainly composed of Quarts, Plagioclase, and Calcite whereas Smectite was detected as a tracer material and there was no record of Pyrite. As both Smectite, and Pyrite minerals were not the major components in the tested samples, the mineralogical influence in the disintegration was minimum in the slaking processes of these rock materials. The presence of soluble calcareous minerals in rock materials such as marble, limestone and dolomite is also subjected to slaking under the slaked conditions [32,33]. Even though dolomite was contained in the tested materials, a small quantity was recorded between 3.5 % and 5.9 % and could not expect a significant contribution to the slaking of the materials. Based on the mineralogical analysis, the tested materials exhibited mineral compositions that displayed low degradability. Consequently, these materials demonstrated a reduced susceptibility to slaking when subjected to the applied pressure and environmental conditions.

### 4.2 Index properties

**Fig. 5:**
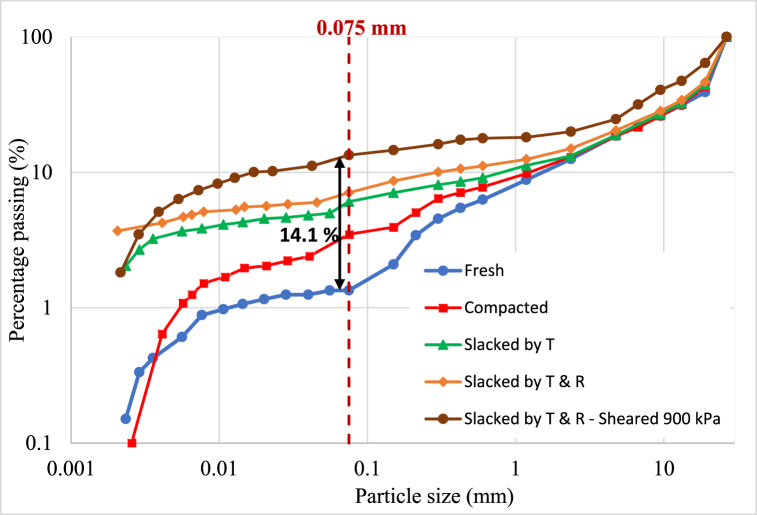
Gradation curves for fresh, compacted, and slaked material and sheared materials after slaking.

Grain size is highly affected on slaking such that materials with high clay contents rapidly respond to the triggering conditions [34]. Therefore, sieve analysis test was conducted on fresh, compacted, both types of slaked material, and sheared slaked materials. Fig. 5 depicts the gradation curves of slaked material, along with the respective comparison with fresh and compacted material. The gradation curves illustrate the breakage of larger particles during compaction and slaking resulting in an increase in finer fraction. Moreover, the particle breakage is greater in material slaked by both temperature and rainfall compared to that of material slaked only by temperature. This signifies the effect of both drying and wetting cycles on slaking process of the materials [35]. Quantitatively there was an increase of 5.74 % and 7.3 % of finer fraction (particles finer than 75 μm) of the materials in slaked by temperature and slaked by temperature and rainfall than the fresh materials respectively.

A significant increase of fines was recorded in the slaked and sheared materials. It was 14.13 % increase to the fresh materials and signifies the triggering action of compaction and shearing on particle breakage of the slaked materials. Disintegration ratios (DR) of the above samples were determined to quantify the fragment size distribution before and after slaking [36]. The range of DR values presents 0 to 1, where 0 indicates low rock durability and 1 indicates the highest durability in terms of prone to slaking. The DR values of the Fresh, compacted, slaked by T and, slaked by T & R indicate 0.376, 0.387, 0.395, 0.410 respectively. The gradual increase of DR values reveals the effect of triggering actions of slaking particularly, the importance of repeated drying and wetting conditions on the samples. The highest DR of 0.592 was recorded in the slaked materials which was subjected to direct shear test. This material has been rapidly disintegrated due to the compaction and shearing after the slaking process. This indicates that materials gradually weaken when subjected to repeated drying and wetting cycles under overburden pressure, making them more prone to slake with minor external impact.

The Atterberg limits provide a useful indication of the properties of the tailing materials, particularly the plasticity index indicates the resistance to liquefaction. The results on Atterberg limits in Table 4 highlight that there was no significant change during compaction, slaking, and shearing as the plasticity index stayed between 11 and 14%. The result further justifies the fact that the plasticity index of soil has not significantly affected by compaction, slaking and shearing, as the process does not involve any mineralogical changes. Similarly, the particle density was not affected by compaction, slaking and shearing, and constantly stayed at 2.66–2.67 g/cm^3^.

**Table 4:** Summary of Atterberg limits of slaked material.

Material type	Liquid limit (%)	Plastic limit (%)	Plasticity index (%)
Fresh	23.43	12.17	11.26
Compacted	26.91	13.55	13.36
Slaked by T	27.04	13.22	13.47
Slaked by T & R	28.01	13.82	13.89

### 4.3 Hydraulic conductivity

The constant head permeability test results in Table 5 illustrate the hydraulic conductivity values of fresh, compacted, slaked by temperature, and slaked by both temperature and rainfall material at the water temperature of 25 °C. The increase of fine content resulting from the slaking process induces a self-sealing effect, leading to a reduction in permeability characteristics [37]. The fine particles effectively occupy the interstitial spaces between the skeletal grains, reduction in the soil porosity, and refining of the pore structure of materials, consequently causing a marginal reduction in the permeability of slaked materials [38]. Therefore, the hydraulic conductivity was reduced by 3.65E-05 m/s in compacted material than that of fresh material. Similarly, the hydraulic conductivity was further reduced by 1.17E-05 m/s and 7.96E-06 m/s due to slaking by temperature and slaked by temperature and rainfall, respectively.

**Table 5:** Summary of hydraulic conductivity.

Material type	Hydraulic conductivity (m/s)
Fresh	6.49E-05
Compacted	2.84E-05
Slaked by T	1.67E-05
Slaked by T & R	8.74E-06

### 4.4 Shear strength

**Fig. 6:**
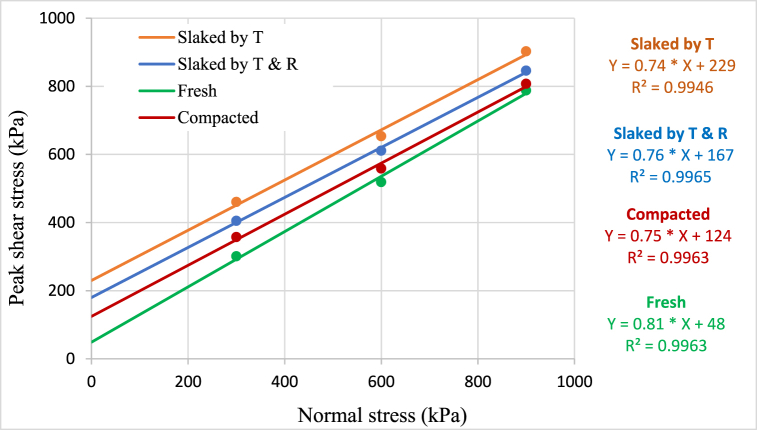
Mohr-Coulomb failure envelop for peak shear stress failure criteria.

**Fig. 7:**
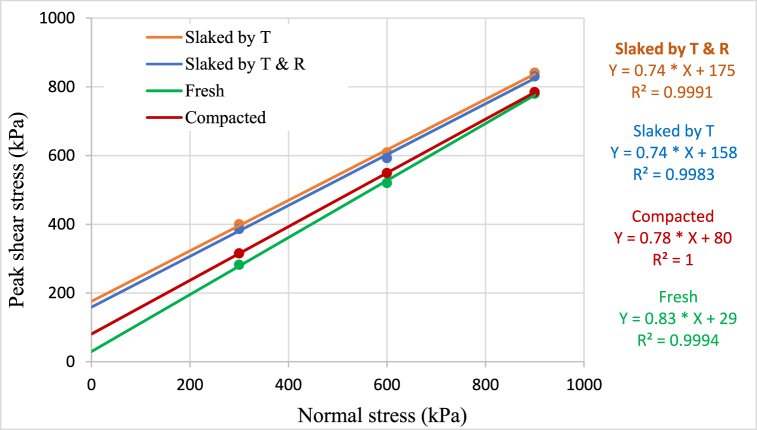
Mohr-Coulomb failure envelop for residual shear stress failure criteria.

Direct shear tests were conducted on fresh, compacted, and slaked material under the vertical stresses 300, 600 and 900 kPa using the large direct shear apparatus. Table 6 summarises the direct shear test results, and Fig. 6, Fig. 7 depict the Mohr-Coulomb failure envelopes for peak and residual shear stress failure criteria, respectively.

**Table 6:** Summary of direct shear test results for fresh, compacted, and slaked material.

	Peak shear		Residual shear	
	Cohesion (kPa)	Friction angle (^0^)	Cohesion (kPa)	Friction angle (^0^)
Fresh	48.85	39.06	29.56	38.75
Compacted	124.44	36.87	95.96	36.62
Slaked by T	229.74	36.41	163.04	35.99
Slaked by T & R	182.15	35.97	175.67	35.90

Results convey that the cohesion increases during compaction and slaking, compared to the fresh material, as a result of particle breakage. The friction angle has decreased in the compacted and slaked materials such that an approximately 2° drop of friction angle was observed in the compacted materials than the fresh materials. Similar results were obtained in both types of slaked materials with respect to the peak and residual states. The cohesion and friction angle related to peak shear parameters are marginally higher than that of residual shear parameters. Sharma et al. [12] noted a consistent trend of decreasing friction angles with an increasing number of wetting-drying cycles on crushed mudstones. However, it is important to emphasize that the mineral composition of the material exhibits significant sensitivity to alterations in shear strength parameters [39]. The influence of mineralogy on shear strength properties following the slaking process is elucidated in the findings of Vlastelica et al. [40]. His observations highlighted the impact of calcium carbonate content on the alteration of shear strength parameters, demonstrating an increase in values when weathered materials contained higher levels of calcium carbonate.

## 5 Discussion

The process of slaking is very complex, and it is assumed due to the disruption of diagenetic bonds and the release of the stored energy of rock materials [41]. There is an effect of the overall shape and surface roughness of some materials on slaking, particularly in the presence of clastic sedimentary rock materials [42]. However, this effect may not be applicable to all types of materials and requires comprehensive analysis across different material types.

Slaking occurs under different conditions and the slaking process is triggered when [43,44].

• exerted stresses are being increased,
• the trapped air compress within the soil particles and lumps, particularly in soils containing expansive soils,
• soil is immersed in water,
• materials are subjected to drying and wetting cycles.

Therefore, more accurate and reliable results on estimating the susceptibility to slaking of materials can be obtained by simulating the effects of immersion, pressure, wetting and drying on the testing materials for an adequate time. However, the existing standard tests of estimating the slaking have many drawbacks. These test methods do not cover all these requirements within a single test procedure therefore, effects of all triggering actions could not be evaluated by conducting one test. This deficiency could be overcome by the introduced test procedure in this study since it applies all triggering actions to slake the test materials.

Confining pressure is not significantly modelled in many slaking tests; instead, tumbling effect is employed which does not generally exist in the field [45,46]. This study introduced 900 kPa of overburden stress to represent approximately 40 m–45 m thick overlay in a confined cell which can be adjusted as per the requirements. The overburden pressure was maintained throughout the testing time. Initially, compacted materials were left over for 24 h with vertical stress to ensure uniform pressure distribution on the compacted samples. Samples were then saturated for 1–2 days to replicate the field water saturation process.

Weathering conditions were applied to the samples within an environmental chamber under two test scenarios: ‘temperature and humidity cycles' and ‘wetting and drying cycles'. This approach effectively induces the slaking mechanism by regulating temperature, moisture, and humidity. Adequate immersion is a significant factor to trigger the slaking process. This study introduced fortnight wetting and drying cycles thereby, the compacted materials were subjected to fully saturated condition for a week then, drying state in the following week. The wetting and drying cycles play an important role in softening of rock materials and are most effective on slaking [47,48]. When the materials are being wetted after drying cycle the entrapped air pressure increases between the particles and causes degradation. It has been observed that degradation increases with increasing drying period of the materials [49]. Therefore, wet and dry conditions were applied as repeated cycles to simulate in-situ weathering behaviour.

A “linear variable displacement transducer (LVDT)” and a digital scale were attached to the loading plate of each cell to measure the potential collapse or sudden volume change when the slaking is in progress. The results obtained indicate that there is an insignificant volume change in the tested samples during the slaking process, which can be attributed to the compositions of the materials studied in this research. Nevertheless, the proposed method recommends fixing the LVDT and continuously monitoring it, as this is essential for measuring the progression of slaking over time.

The standard test methods generally measure the slaking and indicate it as an index however, they do not measure the relative variations of the properties due to degradation during the slaking process. This study emphasises the importance of conducting the physical and strength property tests on the slaked materials including the XRD analysis. The physical and strength properties of the slaked materials can be applied to explain the mechanical behaviour of the slaked materials under different circumstances. Determination of the mineralogical composition is also essential, since the variation of properties can be attributed to differences in the mineralogical compositions, clay fraction and, rock fragments susceptible to slaking [50]. Therefore, the developed laboratory test procedure can be introduced as a comprehensive and reliable method to evaluate the susceptibility to slaking and to determine the slaking-induced changes in geotechnical properties of rock materials.

## 6 Conclusion

This study was undertaken to devise an innovative laboratory-scale testing approach for assessing the slaking potential of materials. The aim was to address the shortcomings identified in existing test methods, including inadequate sample size representation, limited testing duration, absence of slaking condition simulation, and the influence of the test performer. The experimental methodology was meticulously devised to encompass all potential triggering factors mentioned below that contribute to the slaking process,

• Applying overburden pressure similar to the in-situ conditions of the initial location of the materials.
• Fully saturation of the test samples to replicate the actual field water saturation process.
• Replication of in-situ weathering conditions by subjecting samples to temperature and humidity cycles, as well as wetting and drying cycles.

Recognizing the significance of duration in inducing slaking, the experimental setup operated for a continuous six-month period, offering flexibility for both short-term and long-term assessments as needed. Additionally, this study underscores the importance of determining the physical properties of slaked materials to unveil the consequences of slaking. This entailed the evaluation of material properties, including mineralogical analysis, both before and after the slaking process.

The impact of the slaking process was most notable in the cohesion of the materials, while other properties exhibited minor variations due to the limited extent of degradation during slaking. The mineralogical composition of the tested materials displayed less responsiveness to slaking triggers, resulting in reduced material degradation. Therefore, it strongly recommends the incorporation of XRD analysis, as it provides valuable insights into the mineralogical responses of the test materials to the slaking process.

## Data availability statement

Data will be made available on request.

## CRediT authorship contribution statement

**Chaminda Gallage:** Writing – review & editing, Supervision, Project administration, Methodology, Investigation, Funding acquisition, Formal analysis. **Shiran Jayakody:** Writing – review & editing, Methodology, Formal analysis. **Tharindu Abeykoon:** Writing – original draft, Methodology, Investigation. **Dareeju Biyanvilage:** Writing – review & editing, Resources. **Jay Rajapakse:** Supervision.

## Declaration of competing interest

The authors declare that they have no known competing financial interests or personal relationships that could have appeared to influence the work reported in this paper.
